# Transactions of the Chicago Gynæcological Society

**Published:** 1879-04

**Authors:** T. D. Fitch


### Society Reports.

### ARTICLE VII.

TRANSACTIONS OF THE CHICAGO GYNÆCOLOGICAL SOCIETY. Fourth Meeting, January 31st, 1879. T. D. FITCH, M.D., in the chair.

Dr. E. C. Dudley read a paper on Lacerations of the Cervix Uteri (published in the March number of the JOURNAL AND EXAMINER).

### DISCUSSION.

DR. JACKSON: I freely indorse all that has been urged as to the frequency and pathological importance of the lesion under consideration. As an illustration of the grave symptoms it is capable of producing, I may mention the case of a lady who was sent to the Woman's Hospital of the State of Illinois from Iowa some three years ago. The injury was the result of parturition attended by the birth of an unusually large child. She had a slow and imperfect recovery; and, although able to resume her household duties after a few weeks, she soon found herself suffering so greatly that she took to the bed, and was wholly bedridden for more than two years. She was brought on a bed to the hospital.

On examination, I found a bilateral laceration of the uterine cervix extending on each side to the vaginal junction. The separated lips were rolled outwards, and the thickened, everted mucous lining was studded with follicular cysts and bled upon the least pressure. Her general health seemed completely broken down.

After suitable preparation, the operation of restoring the parts was successfully performed. She rapidly regained her health, and, after a few months, returned to her home, where she was a subject of wonder to her friends.

I have operated 19 times for this malady, with this result: One case was followed by a rather severe attack of pelvic cellulitis, but the patient recovered and the operation succeeded; in another case there was hæmorrhage requiring the vaginal tampon; in three cases the parts failed to unite, in two completely, in one union was partial, In two of these a second operation was successful; the third declined further treatment.

My mode of operation differs in some details from that of Emmet, as given by himself and by the essayist. I do not make use of Sims' speculum for exposing the parts, nor of his little hook for steadying them. I place the patient recumbent on a table, and after etherization, she is drawn down so far that the hips project several inches beyond it. She is now put in the lithotomy position, and this retractor, which I have shown you, and which is a simple modification of that devised by Sims for vesico-vaginal fistula, is introduced and the perineum drawn strongly downward and held by one of the assistants. In this position, and by this means, the uterus falls naturally *towards* the vulva, and not *from* it, as it does in the position recommended by Sims.

Again, in order to curtail the movements of the cervix, I am in the habit of passing a stout silk ligature through both lips of the cervix at a point which marks the center of the os. The two ends of this can be held by an assistant quite out of the way of the operator. To steady the lips while they are being pared, and while the needles are being passed, I use this double tenaculum, which has four times as much holding power as Sims' hook. Indeed, I have found this latter instrument quite insufficient to steady the parts while the needles are being passed through the dense walls of the cervix. I pare the surfaces which are to be united liberally, and prefer for the purpose those scissors known as Byford's. I use thick round needles, such as are employed for the closure of vesico-vaginal fistula, and silver wire for the sutures.

Instead of removing the sutures at the end of the week, as recommended by Emmet, I am in the habit of permitting them to remain 9 or 10 days.

Since I have adopted these changes, I have been enabled to perform the operation with much more facility, and in much shorter time than before.

DR. BYFORD read a letter from Dr. Newman, of Denver, on the subject, which may be found in the present number under the head of correspondence.

DR. SAWYER thought the profession were less likely to err in overlooking the lesion in question than in attributing too great a rôle to the accident as a cause of real discomfort and injury. He instanced a case of a lady whom he had examined recently for the purpose of finding out if she was pregnant, and was surprised to find a marked extropion of the cervix uteri. Upon inquiry, there were absolutely no subjective symptoms of the injury, though three years had elapsed since her last pregnancy.

DR. JONES mentioned the rarity of laceration in his own obstetric practice, and the entire immunity from consequent inconvenience in a recent and pronounced case.

He also called attention to certain questions of diagnosis depending upon the direction in which the long axis of the cervical ellipse might lie, and the doubt whether a true follicular degeneration of the *external* cervix might not sometimes occur, determining a less value to the sign when present.

